# Supplementary material for: Influence of the Interactions at the Graphene–Substrate Boundary on Graphene Sensitivity to UV Irradiation
Source: Materials (Basel). 2019 Nov 28;12(23):3949. doi: 10.3390/ma12233949 (PMC6926663; doi:10.3390/ma12233949)
Supplement: Supplementary file 1 [file materials-12-03949-s001.pdf]

materials

MDPI

Supplementary Materials

Influence of the Interactions at the Graphene–Substrate Boundary on Graphene Sensitivity to UV Irradiation

Dorota Nowak [1,*], Marian Clapa [1,2], Piotr Kula [1], Mariusz Sochacki [3], Bartlomiej Stonio[3], Maciej Galazka [2], Marcin Pelka [2], Dominika Kuten [2] and Piotr Niedzielski [1]

Table S1. State of the substrates surface before transfer and the quality of graphene on final substratum.

Substratum	Substratum + Graphene
**Quartz Glass**	
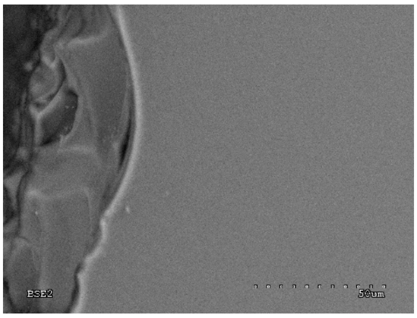	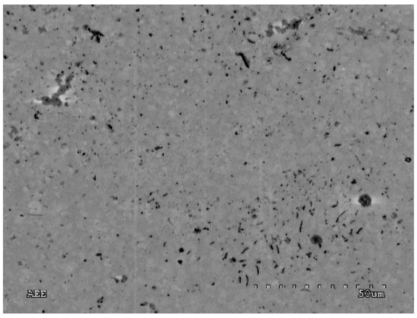
**PTFE**	
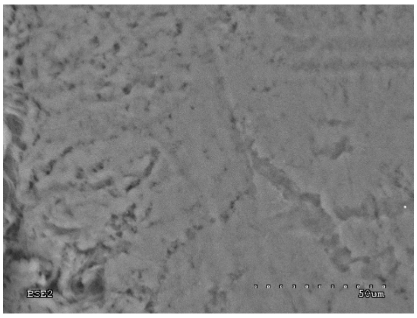	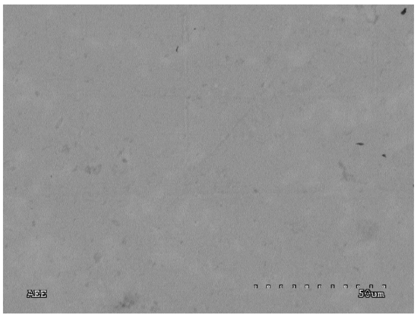
**kapton**	
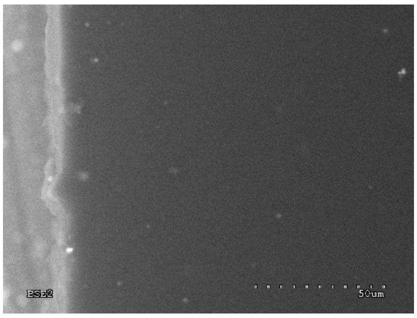	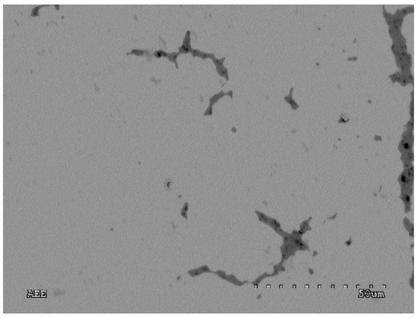
**PEN**	

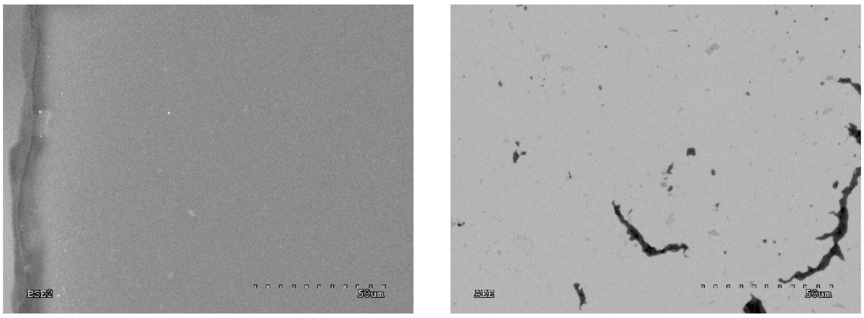

Raman mapping was performed for graphene transferred on Si/SiO_2 substrate before UV irradiation to check the homogeneity of graphene. Figure S1 exhibits Raman I_{2D}/I_G mapping. Figure S2 shows the fitting of 2D band for trilayer graphene.

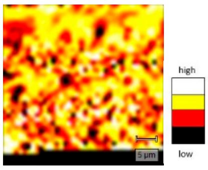

Figure S1. Raman $I_{2D/G}$ imaging of graphene on Si/SiO_2.

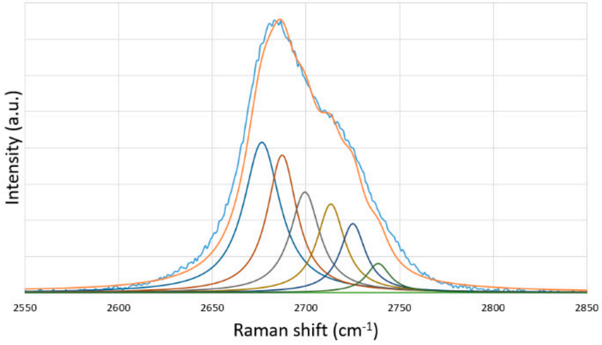

Figure S2. Fitting of 2D band for trilayer graphene.

© 2019 by the authors. Submitted for possible open access publication under the terms and conditions of the Creative Commons Attribution (CC BY) license (http://creativecommons.org/licenses/by/4.0/).
